# Renal scarring in children with febrile urinary tract infection

**DOI:** 10.1016/j.jped.2024.10.011

**Published:** 2025-01-31

**Authors:** Arife Uslu Gökceoğlu, Nesrin Taş

**Affiliations:** a Alanya Alaaddin Keykubat University, Faculty of Medicine, Department of Pediatric Nephrology, Antalya, Türkiye; b Ankara Training and Research Hospital, Department of Pediatric Nephrology, Ankara, Türkiye

**Keywords:** Dimercaptosuccinicacid scan, Renal scarring, Renal bladder ultrasonography, Urinary tract infection

## Abstract

**Objective:**

The authors aim to evaluate characteristics of children with fUTI and results of renal bladder ultrasonography (RBUS) and late dimercaptosuccinicacid (DMSA) scan.

**Methods:**

This study is designed as retrospective analysis of RBUS and DMSA reports of children with fUTI. Age, gender, number of fUTI, presence of constipation and vesicouretheral reflux (VUR) were recorded.

**Results:**

The study included 160 children with fUTI with a median age of 7 years (6 months 18 years old). The majority of children in this study were girls (86.3 %), older than 60 months (73.1 %) and had one episode of fUTI. The recurrence rates of UTI were similar in both girls and boys. The total rate of constipation was 21.9 %. The rate of renal scarring on DMSA was 16.9 %. The rates of renal scarring were similar at three age groups and both genders. The rate of renal scarring was higher in children with recurrent UTI compared to those with one episode of fUTI (26.4 % and 12.5 %, respectively; *p* = 0.04). The rate of constipation in children with renal scarring and normal DMSA was similar (*p* = 0.07). The rate of trabeculation and thick bladder wall was higher in children with renal scarring at DMSA than children with no renal scarring (*p* = 0.03).

**Conclusion:**

The present study demonstrated that 16.9 % of children with fUTI had renal scarring. The rates of renal scarring were similar in both gender and age groups. Children with recurrent UTI and abnormal bladder results at RBUS had higher rates of renal scarring.

## Introduction

One of the most common bacterial infections in children are urinary tract infections (UTI) and the incidence of UTI varies according to age, gender and circumcision status of child.^1^ The incidence of UTI in boys is 5.3 % for the first 6 months of age and decreases with age to 2 % for ages between 1 and 6 years. The incidence in girls is 2 % for the first 6 months and increases with age around 11 % for the ages between 1 and 6 years.^2^

UTI without fever is localized to bladder and easily treated. In contrast, children with fever have increased probability of kidney involvement, increased risk of underlying nephrourologic abnormalities and a greater risk of renal scarring.^3^ Renal scarring due to UTI has many long-term morbidities such as chronic kidney disease (CKD), hypertension and preeclampsia.^4^^,^^5^ The prevalence of renal scarring after febrile UTI was reported as 12%– 47 % that varies among studies and unrelated to age.^6^, ^7^, ^8^, ^9^, ^10^ It is important for clinician to know which children has higher risk of renal scarring. Knowledge of children with higher risk of renal scarring may prevent late diagnosis and on the other hand knowledge of children with lower risk of renal scarring prevents further unnecessary imaging. The objective of this study is to evaluate the characteristics and late dimercaptosuccinic acid (DMSA) scan results of children with febrile urinary tract infection (fUTI).

## Material-method

The present study is designed as a retrospective analysis of reports of children with fUTI, based on RBUS and DMSA data. This study was conducted in accordance with the principles set forth in the Declaration of Helsinki. Approval of the study was granted by the Ankara Training and Research Hospital Ethics Committee (07.06.2021/634). In order to be included in the study, patients had to meet the following criteria: they had to be aged ≤ 18 years and have a history of febrile bacteriologically proven UTI. UTI was diagnosed by urine culture. Febrile UTI was determined as growth of a single uropathogene at urine culture with axillary fever > 38°. Patients with congenital anomaly of kidney and urinary tract (CAKUT), chronic renal disease, cystic renal disease, renal agenesis, neurogenic bladder, renal hypoplasia were excluded.

At the time of referral to the Pediatric Nephrology department, the following variables were recorded: age, gender, number of fUTIs and presence of constipation. The children were classified into three age groups: Group 1 included children under 24 months of age, Group 2 comprised children between 24 and 60 months of age, and Group 3 consisted of children between 60 months-18 years. A diagnosis of constipation was made if parents reported that their children had hard stools passed fewer than three times per week and exhibited signs of stool retention upon rectal examination. A recurrent UTI was defined as two or more fUTIs.

The RBUS was conducted at the time of the UTI, and children with results of CAKUT, renal hypoplasia, and renal cysts were excluded from the study. A second investigation with RBUS into renal scarring was undertaken 30 days after the DMSA, and these results were included in the study. A DMSA scan was conducted at least four to six months following the initial diagnosis of fUTI to examine renal scarring. Investigations undertaken for reasons unrelated to UTI were excluded from the study.

All patients underwent both planar imaging (anterior and posterior) and single-photon emission computed tomography (SPECT). The following observations were made split renal function, functional size of the kidneys, and renal scarring. A renal scar was defined as a loss of functional tissue in at least two directions. A significant loss of renal function was defined as a functional difference of 10 % or more between the two kidneys, allowing for a measurement error of up to 10 %.^11^ Abnormal function on DMSA was defined as a differential function of < 45 %.

The results of the RBUS conducted six months after the initial UTI were documented. The kidneys were evaluated according to standard criteria, including renal length, echogenicity, the presence of hydronephrosis, corticomedullary differentiation, and the regularity of the cortical outline. The definition of scarring on ultrasonography was based on the criteria proposed by Barry et al.^12^ (1) Proximity of sinus echoes to cortical surface; (2) Loss of pyramids; (3) Irregularity of outline; (4) Loss of definition of capsular echo; and (5) Calyceal dilatation. Furthermore, the presence of trabeculation and a thick bladder wall on ultrasonography was also recorded. A bladder wall thickness exceeding 3 mm was defined as a thick bladder wall. Additionally, children who underwent voiding cystourethrography (VCUG) were also recorded to evaluate the presence of vesicourethral reflux (VUR).

The data were analyzed using the SPSS, version 26.0. The categorical data were presented in numbers and percentages and evaluated using the Chi-square test. Medians and ranges were used to present continuous data, and nonparametric tests were used for evaluation. The comparison of groups was evaluated using the Student *t*-test. A *p*-value of <0.05 was considered as statistically significant.

## Results

A total of 511 children with DMSA and RBUS results were analyzed between January 2014 and December 2020. The study included 160 children who met the inclusion criteria. The baseline characteristics of patients at the time of referral to Pediatric Nephrology department are presented in Table 1. The majority of the children participating in the study were female (*n* = 138, 86.3 %) and had one fUTI (*n* = 107, 66.9 %). Forty-nine of the female participants (35.5 %) and four of the male participants (18 %) exhibited recurrent UTI (*p* = 0.07). The total rate of constipation was 21.9 % in our study group.

**Table 1:** Characteristics of patients (*n* = 160).

Characteristics	
Median age (minimum and maximum age)	7 years (6 months–18 years old)
Age groups	
< 24 months, n (%)	17 (10.6 %)
24–60 months, n (%)	26 (16.3 %)
60 months–18 years, n (%)	117 (73.1 %)
Gender	
Female, n (%)	138 (86.3 %)
Male, n (%)	22 (13.7 %)
Number febrile UTI (fUTI)	
1 fUTI (%)	107 (66. 9 %)
≥ 2 fUTI (%)	53 (33.1 %)
Scarring in DMSA, n (%)	27 (16.9 %)
Unilateral	25 (15.6 %)
Bilateral	2 (1.3 %)
Scarring in RBUS n (%)	3 (1.8 %)
Trabeculation and/or increased thickness in bladder wall, n (%)	11 (6.9 %)
History of constipation, n (%)	35 (21.9 %)
Girl	32 (20 %)
Boy	3 (1.9 %)

UTI, Urinary tract infection; DMSA, Dimercaptosuccinic acid scan; RBUS, Renal bladder ultrasonography.

A total of 27 children (16.9 %) exhibited evidence of renal scarring on DMSA imaging. Twenty-five children exhibited unilateral renal scarring, while two children displayed bilateral renal scarring. Fifteen patients (9.3 %) exhibited renal scarring alone, while 12 patients (7.5 %) displayed both abnormal differential function and renal scarring. The characteristics of patients with and without renal scarring on DMSA are presented in Table 2. The rates of renal scarring were higher in children with recurrent UTI and RBUS results indicative of bladder trabeculation and thickening of the bladder wall. A comparative analysis was conducted between three age groups of patients with regard to the incidence of recurrent urinary tract infections (UTIs), the presence of renal scarring, and the results of RBUS (Table 3). The rate of renal scarring and rate of recurrent UTI were similar between three age groups (Table 3).

**Table 2:** Characteristics of children with renal scarring on DMSA.

Variables	Scar absent *n* = 133	Scar present *n* = 27	p
Mean Age (years)	7.1 ± 3.9	6.7 ± 2.7	0.55
Sex			
Girl (*n* = 138)	116 (84 %)	22 (16 %)	0.48
Boy (*n* = 22)	17 (77 %)	5 (23 %)	
One episode fUTI (*n* = 107)	94 (87.5 %)	13 (12.5 %)	
Recurrent UTI (*n* = 53)	39 (73.6 %)	14 (26.4 %)	0.04 *
Trabeculation and thick bladder wall, n (%)	5 (3.7 %)	6 (22 %)	0.03 *
Constipation	25 (18.8 %)	10 (37 %)	0.07

DMSA, Dimercaptosuccinicacid scan; UTI, Urinary tract infection; fUTI, Febrile urinary tract infection.

**Table 3:** Comparison of three age groups according to recurrence of urinary tract infection, presence of renal scarring and bladder trabeculation.

Variables	Group 1 (< 24 months) *n* = 17	Group 2 (24–60 months) *n* = 26	Group 3 (60 months–18 years) *n* = 117	p	95 % CI
Rate of UTI Recurrence, n (%)	4 (24 %)	9 (35 %)	40 (34 %)	Group 1 and 2: 0.44	−0.39–0.17
				Group 1 and 3: 0.36	−0.34–0.13
				Group 2 and 3: 0.96	−0.20–0.21
Presence of renal scarring *n* = 27 (%)	2 (11.7 %)	2 (7.6 %)	23 (19.6 %)	Group 1 and 2: 0.67	−0.15–0.23
				Group 1 and 3: 0.38	−0.26–0.10
				Group 2 and 3: 0.71	−0.24–0.01
Bladder trabeculation and thick bladder wall *n* = 11	3 (17.6 %)	0 (0 %)	8 (6.8 %)	Group 1 and 2: 0.08	−0.02–0.37
				Group 1 and 3: 0.28	−0.09–0.31
				Group 2 and 3:0.04*	−0.11–(−0.02)

UTI, Urinary tract infection; DMSA, Dimercaptosuccinicacid scan (* *p* < 0.05 is significant).

A total of three children (1.8 %) exhibited renal scarring on RBUS results. Eleven children (6.9 %) demonstrated trabeculation and a thick bladder wall. The prevalence of trabeculation and a thick bladder wall was higher in children with renal scarring (Table 2). A total of 32 children underwent VCUG examination. Of these, 15 (46.8 %) exhibited vesicoureteral reflux (VUR). Among the 15 children with VUR, nine (60 %) demonstrated renal scarring.

## Discussion

In this study, the authors evaluated the characteristics and imaging results of children with fUTI. The majority of children in our study were girls (86.3 %), older than 60 months (73.1 %) and had one episode of fUTI. The recurrence rates of UTI were similar in both girls and boys. The total rate of constipation was 21.9 % in our study group. The rate of renal scarring was 16.9 %. The rates of renal scarring were higher in children with recurrent UTI and RBUS results indicative of bladder trabeculation and thickening of the bladder wall. The rate of renal scarring did not differ according to the age, sex and presence of constipation.

Children with fUTI have a greater risk of renal scarring.^3^ Late DMSA scanning should be performed to evaluate the presence of permanent renal scarring after UTI.^13^ The rates of renal scarring in the literature vary from 10 % to 40 % depending on the study design. In one study, the rate of renal scarring in children with upper urinary tract infection was 13 %.^14^ Zaki et al.^15^ reported that persistent paranchymal defects on DMSA was observed at the rate of 38 % in children with one episode of fUTI. The overall rate of renal scarring in our study was 16.9 %, which is similar to that reported in the literature. Of course, there are many questions about the factors that cause renal scarring. One of these questions is whether the gender of the children may affect the prevalence of renal scarring. There are different reports in the literature about gender and renal scarring. In the general population, the prevalence of UTI is higher in women than in men in all age groups except the elderly. The majority of participants (90.4 %) in both RIVUR and CUTIE were female.^16^^,^^17^ Despite the higher prevalence of UTI in females, renal scarring was similar in males and females in the RIVUR study.^16^ In another study, girls were more likely to develop APN and renal scarring than boys.^15^ Silva et al.^18^ reported that boys had higher rates of renal scarring. However, this study included children with VUR and boys in the study group had higher grades of VUR. The present study did not identify any significant differences in the incidence of renal scarring according to the sex of the children. However, it should be noted that a limitation of the study is the lack of VCUG results for all children included in the study.

There is also an association between age, recurrence of UTI and renal scarring. Renal scarring is common at younger ages (<12 months) of UTI onset.^19^^,^^20^ The established risk factors for recurrent UTI are age, sex, race and circumcision status.^18^^,^^21^^,^^22^ The rates of renal scarring were similar between the three age groups in our study. The predominance of children older than 60 months (74.1 %) in our study and the similarity between the recurrence rates of UTI in three age groups may be a factor for the similarity of the renal scarring rates. The rate of renal scarring in children with recurrent fUTI was higher than that of one episode of fUTI in our study. The recurrence rate of UTI was similar between both genders. But the similarity of recurrence rate may be the result of small number of males in our study. CUTIE study reported that children without VUR had renal scarring at a rate of 5.6 %.^16^ Bowel and bladder dysfunction (BBD) consists of a spectrum of lower urinary tract symptoms (LUTS) and fecal elimination problems such as constipation and/or encopresis.^23^

Furthermore, constipation is linked to lower urinary tract dysfunction (LUTD), and its management has been reported to be an important tool in the treatment of patients with LUTD.^24^, ^25^, ^26^ It is established that there is an association between recurrent UTI and constipation.^27^ The rate of constipation in children with UTI was reported as 30 %.^28^ In our study the rate of constipation was lower than that reported in the literature. The limitation of our study was that the authors evaluated constipation due to reports of parents as constipation may be unrecognized by parents.

The early identification of children at high risk through the widespread use of ultrasonography enables clinicians to reduce the incidence of renal scarring. RBUS is a non-invasive and sufficiently sensitive method for the evaluation of collecting system dilatation. The procedure is most commonly employed for the assessment of children who have experienced a urinary tract infection (UTI). The rate of trabeculation and a thick bladder wall was higher in children with renal scarring in the present study. In some cases, abnormalities in bladder reports identified through RBUS may not be discerned by the clinician. However, the findings indicate that the presence of trabeculation and a thick bladder wall is a significant indicator for evaluating children at an elevated risk of renal scarring.

The present study is limited by its retrospective nature, wide age range group and there is interobserver variability. For all imaging studies performed at the studied institution, radiology reports generated by multiple radiologists were reviewed for the purposes of this study. However, this may represent better real-world clinical experience.

## Conclusion

The present study demonstrated that 16.9 % of children with fUTI had renal scarring on DMSA. The rates of renal scarring were higher in children with recurrent UTI and with the result of trabeculation and thick bladder wall on RBUS. The rate of renal scarring did not differ according to the age and sex of the children.

## Authors’ contributions

All authors contribute to the study conception and design. Data collection and analysis were performed by Arife Uslu Gökceoğlu and Nesrin Taş. The first draft of the manuscript was written by Arife Uslu Gökceoğlu. All authors read and approved the final manuscript.

## Ethics approval

Approval was granted by the Ethics Committee of Ankara Training and Research Hospital (07.06.2021/634).

## Declaration of generative AI and AI-assisted technologies in the writing process

During the preparation of this work the authors used DeepL in order to improve language. After using this tool/service, the authors reviewed and edited the content as needed and took full responsibility for the content of the publication.

## Conflicts of interest

The authors have no relevant financial or non-financial interests to disclose.
